# The Hidden Geometry of Water: Mapping Nanoscale Confinement and Water Properties Inside Peptide–Nucleotide Biomolecular Condensates

**DOI:** 10.1021/jacsau.6c00873

**Published:** 2026-07-17

**Authors:** Ayat Bdarneh, Nadav Amdursky

**Affiliations:** † Schulich Faculty of Chemistry, Technion–Israel Institute of Technology, Haifa 3200003, Israel; ‡ Chemistry, School of Mathematical and Physical Sciences, 7315 University of Sheffield, Sheffield S3 7HF, U.K.

**Keywords:** photoacids, photobases, excited-state proton transfer, biomolecular condensates, liquid–liquid phase separation, microenvironments, time-resolved fluorescence, intrinsically disordered proteins

## Abstract

Biomolecular condensates formed by liquid–liquid phase separation create dynamic, water-rich microenvironments that are essential for cellular organization, yet it is challenging to understand their internal aqueous phase. Here, we introduce Brønsted photoacids and photobases as a new class of environmental fluorescent probes to interrogate the water phase inside biomolecular condensates. Using in vitro condensates formed by intrinsically disordered poly lysine or poly arginine peptides with nucleotides, we combine steady-state and time-resolved fluorescence to resolve the probe’s excited-state proton transfer and subsequent proton recombination dynamics. We show that both photoacids and photobases remain largely solvated within poly lysine-based condensates, with only modest slowing of proton transfer relative to bulk water, consistent with a moderately more viscous aqueous environment. We mainly focus on photoacids, which exhibit strongly enhanced geminate proton recombination, attributed to nanometre-scale confinement of water within the condensates, inducing reflective boundaries for the dissociated proton. By modeling the recombination kinetics, we use the photoacid as a molecular ruler to estimate the dimensions of confined aqueous nanocavities, revealing characteristic maximum radii of ∼6 nm. Altering condensate composition systematically modulates these properties, with ATP- and poly arginine–based condensates displaying denser, less hydrated interiors. These findings establish excited-state proton transfer probes as powerful tools for quantifying nanoscale water confinement in biomolecular condensates, with implications for condensate function and molecular sequestration.

## Graphical abstract

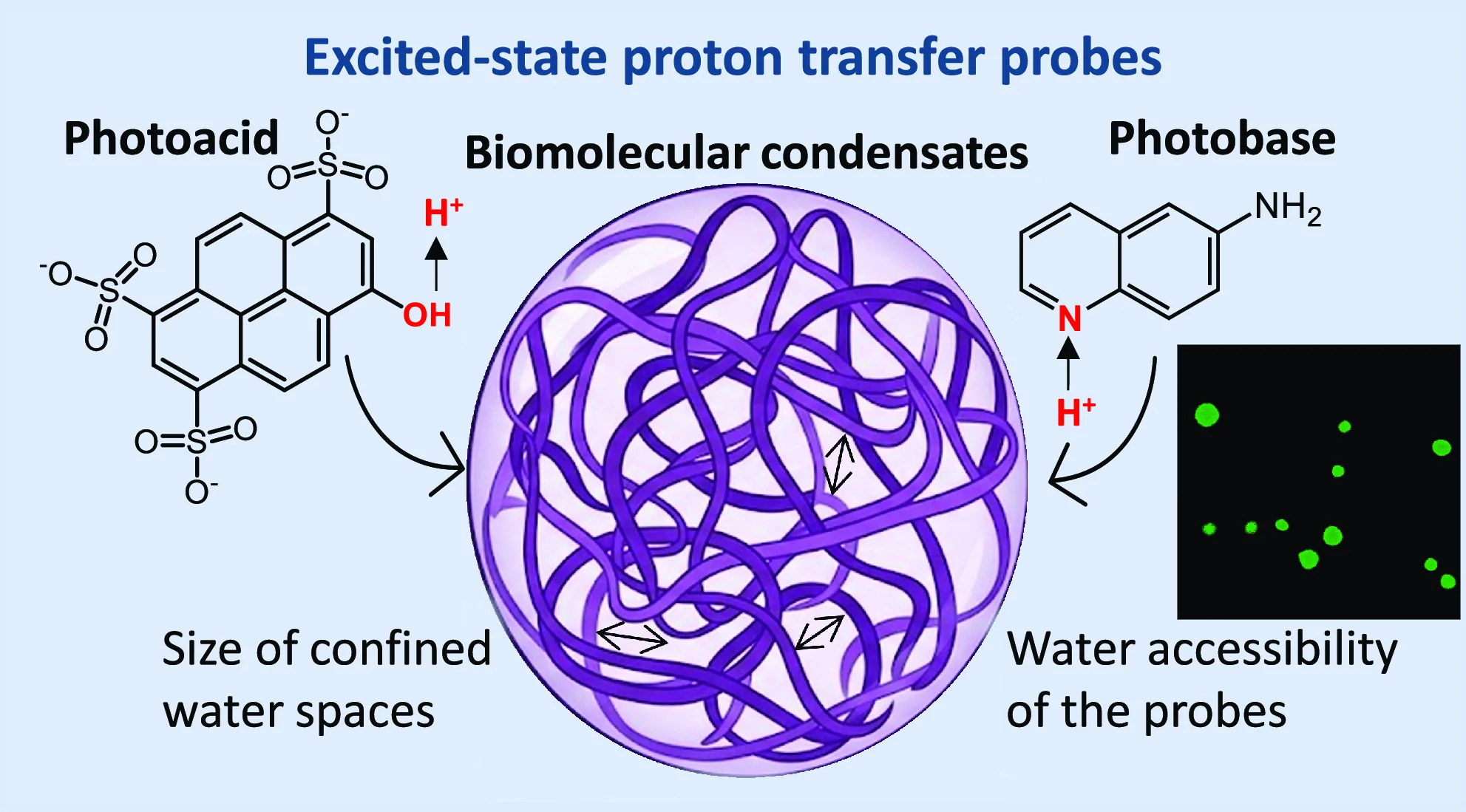

## Introduction

Biomolecular condensates are membraneless assemblies primarily composed of proteins and/or nucleic acids. The formation of these condensates is predominantly driven by a physical process known as liquid–liquid phase separation (LLPS).^1–4^ In LLPS, components spontaneously transition from a state of solubility to a separated condensed state, having two distinct liquid phases: a dense phase, which is highly enriched in the macromolecules, and a dilute phase, where these components are largely depleted.^5^ The dense phase often manifests as spherical and liquid-like droplets.^2^ From a physiological perspective, biomolecular condensates play an important and active role in a wide array of biological processes, which are critical for maintaining cellular organization and provide specialized microenvironments with a dynamic nature.^1–4^ This work aims to introduce a new biophysical approach to understanding the microenvironments within the condensates.

The formation of biomolecular condensates through LLPS is fundamentally governed by a delicate balance of attractive interactions that overcome the entropic penalties associated with demixing.^6,7^ These interactions are typically weak and multivalent, allowing for dynamic and reversible assembly. The multivalent nature of these interactions is critical, as it enables the formation of extensive, long-range intermolecular networks.^8^ Unlike the strong, highly specific interactions that characterize the formation of stable, stoichiometric protein complexes (such as the cytoskeleton or viral capsids), the interactions driving LLPS are generally weak and transient.^1–4^

Several interactions participate in the proteins/nucleic acids self-association and subsequent phase separation. The most common are electrostatic Interactions between the charges of the amino acids and/or nucleic acids. These interactions can be intramolecular or occur through cophase separation of two oppositely charged biomolecules via a process known as complex coacervation.^1–4^ Other interactions include π–π interactions, hydrogen bonds, and even hydrophobic interactions (though they are more common in stable structures).

Here, we use an in vitro model of biomolecular condensates of intrinsically disordered proteins (IDPs), which are formed by complex coacervation from IDPs rich in positively charged residues and nucleic acids.^9,10^ Hence, the assembly of the condensate is primarily driven by electrostatic interactions.^11^ This attraction leads to the aggregation and subsequent phase separation of the mixture into a dense, polymer-rich coacervate phase and a dilute, polymer-poor supernatant phase.

From its name, LLPS separates two liquid phases. Over the years, several biophysical approaches have been employed to decipher the properties of the inner aqueous phase within condensates, including fluorescence-based diffusion measurements, primarily fluorescence recovery after photobleaching and fluorescence correlation spectroscopy, single-particle tracking, small-angle neutron/X-ray scattering, nuclear magnetic resonance, vibrational spectroscopies, and fluorescent dyes. The current understanding is that the water inside condensates remains fluid yet exhibits an apparent slowdown compared to bulk water, with a local microviscosity higher than that of bulk water.^12–16^ One of the earliest observations was made using THz spectroscopy, which was used to characterize changes in the hydrogen-bonding network within protein condensates, concluding that the solvent’s thermodynamic contribution is comparable to that of the protein itself.^17^ THz-calorimetry framework can further distinguish between cavity-wrap and bound hydration water populations, thus demonstrating that LLPS is driven by changes in hydration entropy and enthalpy, highlighting water as an active thermodynamic contributor rather than a passive solvent.^18^ Ultrafast two-dimensional infrared spectroscopy provided a more detailed look at the picosecond hydrogen bond dynamics and structural rearrangement of water molecules within the confined condensate environment, revealing a significant slowdown of water dynamics compared to the bulk solvent.^19^ Raman spectroscopy was also used to probe the altered environment within condensates, which suggested a water activity reduction inside the droplet.^20^ In this work, we use fluorescent probes. To date, the main environmental fluorescent probes that have been used in the study of biomolecular condensates are 6-acetyl-2-dimethylaminonaphthalene (ACDAN) and BODIPY-based probes.^21–24^ The excited state of ACDAN is highly sensitive to the dipolar relaxation (dielectric) of the surrounding solvent, whereas BODIPY acts as a molecular rotor for the microviscosity within the condensates.

Here, we introduce a new family of environmental fluorescent probes for investigating the inner water phase of biomolecular condensates, whose high sensitivity stems from light-triggered excited-state proton transfer (ESPT) processes. These fluorophores function as Brønsted photoacids—undergoing proton dissociation in the excited state and Brønsted photobases—undergoing protonation in the excited state. This unique feature of photoacids and photobases is due to a dramatic change in their p*K*_a_ values between their electronic ground and excited states (further discussion below).^25^ Since the protonated and deprotonated species of photoacids and photobases emit at different wavelengths, it is easy to follow only a specific species or to quantify the ratio between them. Time-resolved fluorescence of the fluorophores allows direct exploration of the water phase surrounding the photoacid or the photobase.^26–30^ In line with previous studies, we also observe that the aqueous phase trapped within the biomolecular condensate is altered from bulk water. We further highlight the use of photoacids, which allows spatial estimation of the aqueous micro/nanocavities within the condensate by using the geminate proton recombination process as a molecular ruler.^31,32^ We find that such aqueous nanocavities have a radius on the nanometre scale. Given the dynamic nature of biomolecular condensates, our approach presents a new opportunity to investigate these trapped aqueous nanocavities. Our findings are important for understanding trapped solutes within biomolecular condensates, which is significant for both their physiological roles and drug-delivery applications.

## Results and Discussion

### The Biomolecular Condensate Models

In this study, we use an in vitro model for biomolecular condensates of IDPs composed of poly lysine (polyK) or poly arginine (polyR) peptides, together with UTP or ATP (Table S1 for molecular structures and Figure S1 for microscopy images of the condensates).^33^ In the polyK system, ATP acts as a multivalent anionic bridge that connects the positively charged lysine residues, driving condensate formation through charge neutralization and ion pairing interactions, i.e., complex coacervation. Phase-diagram mapping revealed that LLPS occurs only within specified polyK and ATP concentration windows (Figure S2), and is highly sensitive to ionic strength; an increase in salt concentration weakens electrostatic interactions, thereby inhibiting coacervate formation.^34^ Replacing ATP with UTP is expected to result in more hydrated and polar condensates than ATP-based ones due to weaker stacking between uracil bases.^35^ Unlike polyK, polyR exhibits stronger interactions with nucleotides due to the presence of the guanidinium group, which enables both electrostatic and cation–π interactions. This enhanced interaction leads to the formation of more viscous condensates, which remain stable at higher ionic strengths than polyK.^33^

For the incorporation of Brønsted photoacids and photobases into the condensates, we chose an acid-containing photoacid—pyranine (HPTS - 8-Hydroxypyrene-1,3,6-trisulfonic acid) and a primary-amine containing photobase—6-aminoquinoline (6AQ) (Table S1 for molecular structures). The integration mechanism of these charged molecules into the complex coacervate is similar to that of charged/polar drugs or other molecules into the aqueous phase of similar coacervates.^36^ The concentration of the photoacid/photobase is nearly 3 orders of magnitude lower than the main components of the biomolecular condensates, hence, they should not have a major role in the condensate structure. With that said, like any other fluorescent probe, they perturb their environment.

### What can be Observed by the Use of Photoacids and Photobases?

The high sensitivity of Brønsted photoacids and photobases to their environment, specifically to the surrounding hydration, stems directly from their photophysical mechanism, also known as a photoprotolytic cycle. This mechanism, which was proposed by Weller and Förster,^37,38^ involves a dramatic change in the p*K*_a_ between its ground state (before excitation) and its excited state (after excitation) resulting from a change in the energy gap between the protonated and deprotonated forms of photoacids/photobases, in what is known as the Förster cycle (Figure S3). For the photoacid used in this study, HPTS, the ground and excited states p*K*_a_ values are 7.4 and 0.4, respectively.^29^ For the photobase used in this study, 6AQ, the ground and excited state p*K*_a_ values are 5.1 and 13.3, respectively.^25^ This change in p*K*_a_ (Δp*K*_a_) of a specific moiety results in a rapid ESPT process, deprotonation for photoacids and protonation for photobases, which is reversible when the molecule returns to its ground state. The use of photoacids and photobases as biophysical probes results from their excited-state processes. For photoacids it is

1 $$ROH\overset{h\nu }{\to }{ROH}^{*}\underset{Rec}{\overset{ESPT}{⇄}}{RO}^{-*}+H^{+}$$

Following the excitation of the photoacid ( $ROH\overset{h\nu }{\to }{ROH}^{*}$ ), the photoacid can dissociate (the ESPT mechanism) with a rate constant of *k*_PT_. In most cases, as in this study, the proton acceptor for the photoacid is the surrounding aqueous phase for the formation of a hydrated proton. Hence, the magnitude of *k*_PT_ is the first important biophysical output for the local environment of the photoacid. If the photoacid is buried within a less-hydrated microenvironment, a highly viscous environment, or adsorbed to the surface of a biomaterial, *k*_PT_ will be low (slow ESPT process), and if the photoacid is fully accessible to the water phase, *k*_PT_ will be large (fast ESPT process), in the order of 1 × 10^10^ s^–1^.^29^ Since the protonated photoacid (ROH*) and the deprotonated one (*RO* ^–*^) emit in different wavelengths, the difference in *k*_PT_ can be estimated by steady-state fluorescence using the ratio between the peaks. However, for a quantitative estimation, there is a need for time-resolved fluorescence, where *k*_PT_ corresponds to the initial decay of ROH*. The change in fluorescent properties as a function of the microenvironment is not unique to photoacids and can be observed with the aforementioned other fluorophores (ACDAN or BODIPY), although through a different mechanism. However, the uniqueness of photoacids lies in the process that occurs after deprotonation, specifically the recombination (Rec in eq 1) with the geminate proton in the excited state. If the photoacid is accessible to the water phase, the dissociated hydrated proton will rapidly diffuse away from the photoacid, resulting in low recombination efficiency. However, two main environmental processes can substantially increase recombination. (1) If the photoacid is next to a surface, it can induce a dimensionality constraint on proton diffusion away from the photoacid that will manifest in the recombination process. (2) If the photoacid is within a constrained microenvironment that restricts proton diffusion away from the photoacid, it will increase the chances of recombination. In a simplified way, the dissociated proton will “bounce back” from the boundaries of the microenvironment and will recombine with the *RO* ^–*^. As will be described below, we can use it to estimate the size of the aqueous microenvironments within the condensates.

The light-triggered events of photobases are

2 $$RN\overset{h\nu }{\to }{RN}^{*}\left(+AH\right)\underset{Diss}{\overset{ESPT}{⇄}}{RNH}^{+*}\left(+A^{-}\right)$$

Following the excitation of the photobase ( $RN\overset{h\nu }{\to }{RN}^{*}$ ), the photobase can protonate via an ESPT mechanism with an association constant of *k*_*a*_. The proton for this process originates from a nearby acceptor (A), which is commonly water. Similar to photoacids, also for photobases, the more exposed the photobase to water, the faster the ESPT (high *k*_*a*_). Since the ESPT process is a short-distance process, i.e., the length of a hydrogen bond, the solvent (water) is the primary acceptor/donor in this process. While a direct ESPT to other chemical moieties is possible, it is unlikely (much less efficient) in the presence of water, though it can be solvent-mediated.^39^ Unlike the extensive study and modeling of the excited-state dynamics of photoacids, the processes for photobases are less explored. While for photoacids, the initial decay of ROH* can be directly correlated to its initial decay, the situation is more complex for photobases. As will be shown below, the decay of RN* is complex, though, its lifetime can be correlated to *k*_*a*_, which also goes hand in hand with the average lifetime of *RNH* ^+*^. While the excited-state dissociation (Diss in eq 2) is theoretically possible, it has not been established as a viable process for biophysical exploration. Hence, unlike photoacids, photobases are primarily used to assess the aqueous environment, specifically the exposure of the photobase to water within the microenvironment, but not to estimate the size of the microenvironment or the dimensionality of proton diffusion.

### Steady-State Fluorescence of Photoacids and Photobases within polyK-UTP Condensates

At first, we used polyK with UTP as a model biomolecular condensate. The successful integration of either HPTS or 6AQ into the condensates was verified by fluorescence microscopy, which confirms probe partitioning into the dense phase (Figure 1a,b). Importantly, no noticeable leaching out of the photoacid/photobase from the condensate was observed. We used the fluorescence microscopy images to estimate the partition coefficient of the dyes into the condensates to be in the order 10–40, which is in line with the partition of charged fluorescent probes within similar condensate systems.^35,40^ The first characterization is steady-state fluorescence of the encapsulated photoacid (HPTS) and photobase (6AQ) within the polyK-UTP condensates (Figure 1c,d). Owing to the different fluorescence peak position of the protonated and deprotonated states of the photoacid/photobase (the deprotonated photoacid, *RO* ^–*^, and the protonated photobase, *RNH* ^+*^, emit in longer wavelengths), the steady state emission spectrum is an important first indication for the ESPT process. For using photoacids and photobases as probes, we should start with a protonated photoacid and a deprotonated photobase. Meaning that for using photoacids, the pH of the solution must be below its ground state p*K*_a_, while for photobases it should be above the ground state p*K*_a_. The pH of the measurement (pH 7.1) satisfies this condition. For the HPTS photoacid, the steady-state fluorescence of HPTS within the condensates is relatively similar to that of the control of solvated HPTS in the same buffer conditions, albeit with a clearly larger normalized ROH* peak (Figure 1c). This finding means that the inner aqueous environment within the condensate does not considerably inhibit the capability of HPTS to undergo ESPT, such as in highly viscous media; however, there is a clear change in the ESPT kinetics (eq 1) that results in a higher ROH* peak (further discussion below). Since UTP is an oxo-acid, we also ruled out the presence of UTP as the cause of the observed change in the steady-state fluorescence both for the UTP concentration used to make the condensates and for much higher concentration (0.1 M), representing the dense concentration of UTP within the condensates (Figure S4a). For the 6AQ photobase, the change in the steady-state fluorescence between 6AQ within the condensates and 6AQ is more noticeable (Figure 1d). There is a major difference between the capability of photoacids to undergo excited-state dissociation and that of photobases to undergo excited-state protonation, which originates from the nature of the hydrated proton in water and the different capability of water to accept or donate a proton. Accordingly, the ESPT of photobases is less efficient than that of photoacids, as can be seen by the fundamental difference in the ratio of *RO* ^–*^/ROH* (Figure 1c) and *RNH* ^+*^/RN* (Figure 1d) of the controls (solvated HPTS and solvated 6AQ, respectively). This property makes photobases very sensitive to the water state of their surroundings. Accordingly, the fundamentally different steady-state fluorescence of 6AQ within the condensates clearly indicates the different aqueous phase of water compared to bulk water (further discussion below). As above, we also ruled out the presence of the UTP oxo-acid as the cause for the observed change in the steady-state fluorescence (Figure S4b). Nonetheless, for both photoacids and photobases, using only steady-state fluorescence cannot resolve the kinetics associated with the ESPT process, which is mandatory for their use as biophysical probes.

**Figure 1:**
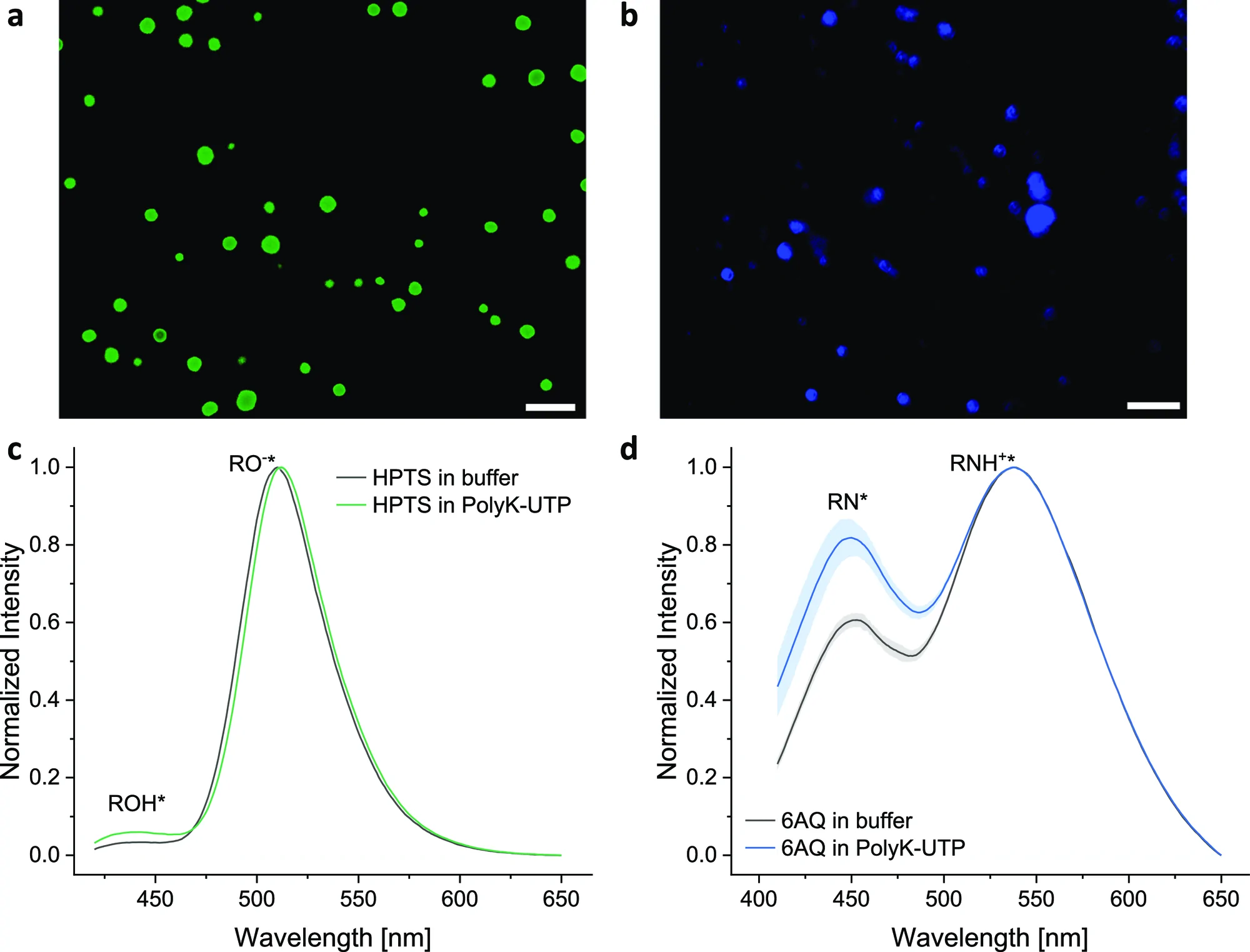
Confocal microscopy images of (a) HPTS and (b) 6AQ within polyK-UTP condensates. The scale bar represents 10 μm. Steady-state fluorescence of (c) HPTS and (d) 6AQ within polyK-UTP condensates compared to solvated HPTS and 6AQ in the same buffer. The shaded area represents the standard deviation of *N* = 3 for HPTS/6AQ in buffer and *N* = 6 for HPTS/6AQ in the condensates.

### Time-Resolved Fluorescence of Photoacids and Photobases within polyK-UTP Condensates

Only via time-resolved fluorescence measurements can we resolve the protonic interplay between the photoacid or photobase and their environment; the processes discussed for eqs 1 and 2.

### Time-Resolved Fluorescence of the Photoacid

Unlike the steady-state fluorescence (Figure 1c), the time-resolved fluorescence of the HPTS photoacid (at the emission wavelength of ROH*) within the polyK-UTP biomolecular condensates is considerably different from the transient of HPTS in the buffer conditions (Figure 2a). As in the steady-state measurements, we ruled out the presence of the UTP oxo-acid as the main cause for the observed difference (Figure S5a). As discussed, several processes happen following the excitation of the photoacid (ROH*). The first is the ESPT (proton dissociation) with a rate of *k*_*PT*_, which manifests in the first hundreds of picoseconds after excitation. As shown in Figure 2a (zoom-in right panel), while there is a difference in the initial decay between solvated HPTS and HPTS within the condensates, it is not as substantial as the differences at longer time scales (>0.5 ns), which are around an order of magnitude in the decay intensity. Since the amplitudes of the longer time scale processes are smaller than the initial amplitude of the ESPT process, such differences are not seen in the steady-state fluorescence. For a more quantitative approach, and owing to the nonexponential nature of the time-resolved decay, we have fitted the transients to a 3-exponential decay analysis (Table 1). As shown in the Table, the first time scale (τ_1_) is relatively similar between solvated HPTS and HPTS within the polyK-UTP condensates. The value of τ_1_ of the solvated HPTS sample is in the same order of magnitude as the known value of *k*_PT_ in water (∼100 ps). Another indication of the similar initial ESPT process between solvated HPTS and condensate-trapped HPTS is the similar rise time of the *RO* ^–*^ species (Figure S6a), which is also in the order of ∼100–150 ps. Following ESPT, several processes influence the proton geminate recombination with a rate of *k*_a_. Such processes involve the fate of the released proton and are influenced by the proton diffusion medium, dimensionality, and increased chances of recombination. As shown in Table 1, there are substantial differences in the later time scales (τ_2_ and τ_3_) between solvated HPTS and HPTS within the condensates, which indicates that the proton geminate recombination of HPTS within the condensates is more substantial within the condensate compared to solvated HPTS. This large magnitude of the “slower” processes results in a 2-fold increase in the averaged lifetime of HPTS within the condensates compared to in solution. Below, we will show how the proton geminate recombination process can be utilized to obtain meaningful information about the aqueous phase microstructure within the condensates.

**Figure 2:**
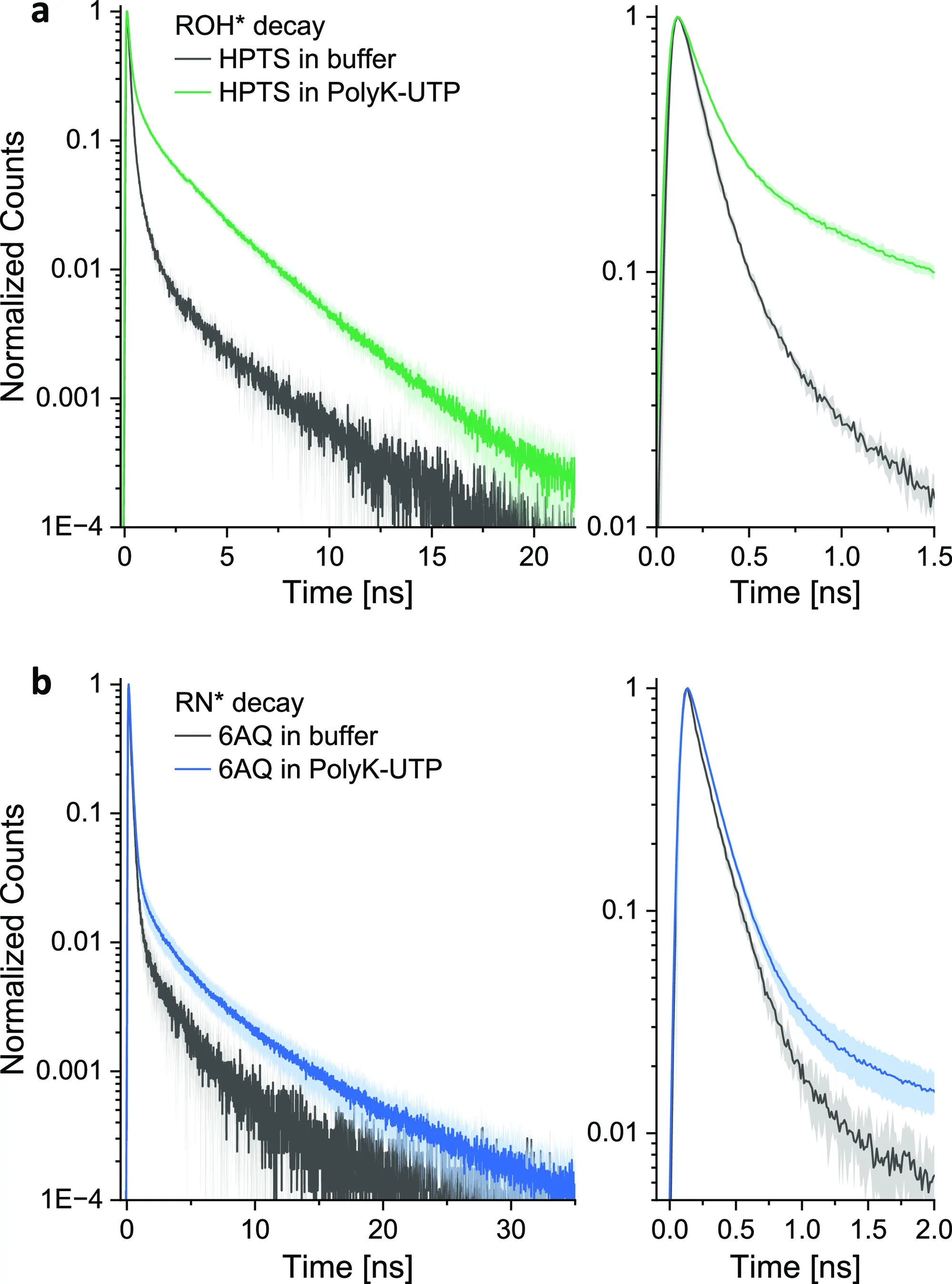
Time-resolved fluorescence of (a) HPTS at the position of ROH* (440 nm) and (b) 6AQ at the position of RN* (450 nm) within polyK-UTP condensates compared to solvated HPTS and 6AQ in the same buffer. The right panels are zoom-in areas of the first 1.5 ns of the decay. The shaded area represents the standard deviation of *N* = 3 for HPTS/6AQ in buffer and *N* = 6 for HPTS/6AQ in the condensates.

**Table 1:** 3-Exponential Fitting for the Fluorescence of HPTS (ROH*) and 6AQ (RN*) within polyK-UTP Condensates and the Overall Averaged Lifetime (⟨τ⟩)^a^

	HPTS photoacid		6AQ photobase	
	solvated	in condensates	solvated	in condensates
*a*_1_	0.64 ± 0.004	0.86 ± 0.01	0.63 ± 0.02	0.96 ± 0.01
τ_1_	0.13 ± 0.01	0.15 ± 0.006	0.16 ± 0.04	0.17 ± 0.006
*a*_2_	0.34 ± 0.005	0.08 ± 0.005	0.37 ± 0.02	0.03 ± 0.01
τ_2_	0.15 ± 0.10	0.80 ± 0.10	0.26 ± 0.02	1.19 ± 0.23
*a*_3_	0.01 ± 0.003	0.06 ± 0.005	0.01 ± 0.002	0.01 ± 0.002
τ_3_	1.61 ± 0.09	2.97 ± 0.16	2.31 ± 0.36	5.15 ± 0.53
⟨τ⟩	0.17 ± 0.01	0.38 ± 0.02	0.21 ± 0.02	0.24 ± 0.02

a The values represent an average and standard deviation of *N* = 3 for HPTS/6AQ in buffer and *N* = 6 for HPTS/6AQ in the condensates. The amplitudes (*a*_n_) are normalised, and the rates (τ_*n*_) values are nanoseconds.

### Time-Resolved Fluorescence of the Photobase

Similar to the use of the photoacid in the biophysical exploration of biomolecular condensates, also for photobases, the time-resolved measurements can result in our understanding of whether the changes in the steady-state fluorescence are related to the fast ESPT process, which indicates the level of solvation of the dye, or to slower processes happening after ESPT. As shown in Figure 2b, the initial fast decay at the first 0.5 ns of the RN* species (right panel of the figure) is relatively similar between solvated 6AQ and 6AQ within the polyK-UTP biomolecular condensates. This finding indicates that the 6AQ photobase is solvated in the aqueous phase within the condensate, and is capable of accepting a proton from the water phase with nearly the same efficiency as in bulk water. Similar to our findings with the HPTS photoacid, also for the 6AQ photobase, the main changes in the time-resolved processes are related to slower processes (>0.5 ns). As before, we ruled out the interaction of the 6AQ photobase with the UTP oxo-acid as the cause for these changes (Figure S5b). Another indication of the similar protonation rate of 6AQ within condensates and that in bulk solution is the relatively similar rise time in the emission wavelength position of *RNH* ^+*^ (Figure S6b). Unlike using photoacids as biophysical probes, where the geminate recombination process is relatively well understood, the slower processes happening following the excited-state protonation of photobases are less understood. One explanation for a higher amplitude fluorescent tail is an enhanced excited-state dissociation process of the newly formed *RNH* ^+*^ (see eq 2). However, this is unlikely to be the cause here since this process should be more efficient in a purely solvated system and not when the photobase is entrapped in a microstructure. The more likely explanation is slower solvent reorganization processes, owing to the altered solvent environment within the condensates. This conclusion about the ability of the water phase to protonate the excited photobase is in line with findings from the ACDAN fluorescent probe, which showed that water dipolar relaxation is often reduced (slower reorientation or lower effective polarity) within biomolecular condensates.^21–24^ Unlike for the use of photoacids, the averaged lifetime of 6AQ within the condensates is merely slightly slower compared to 6AQ in solution (Table 1).

For an interim summary, the use of photoacids and photobases to study the water phase of the polyK-UTP-based condensates led us to conclude that the water phase within the condensates restricts the ESPT process of the dyes due to increased viscosity or slower water dynamics, compared to the ESPT processes in bulk water. However, this change is very modest. Nonetheless, the main differences observed in the time-resolved measurements are in the long-lived fluorescence decay of the photoacids, indicating a change in the proton recombination process, which will be discussed in the next section.

### Photoacids as a Molecular Ruler for the Inner Aqueous Phase within Condensates

As discussed, the long-lived amplitude fluorescent decay of the photoacids’ ROH* species is indicative of the proton geminate recombination process, which is sensitive to the diffusion of the proton around the excited photoacid. When HPTS is solvated in bulk water, the dissociated proton diffuses rapidly from the deprotonated *RO* ^–*^ species, resulting in a very low magnitude fluorescent tail of ROH* at times >3 ns following excitation (Figure 2a). If the photoacid is buried within a hydrophobic pocket, it will influence proton diffusion, and hence, recombination, but it will also exhibit a poor ESPT (proton dissociation),^26–28^ which is not what we observed for HPTS within the condensates. Another possibility for an increase in the recombination process is a dimensionality constraint for proton diffusion, as seen in photoacids tethered to the surface of biological membranes, which might exhibit lateral proton diffusion.^41–44^ However, given the initial fast decay of HPTS in the condensate and the amorphous nature of the LLPS process, there is no preferential low-dimensionality pathway for proton diffusion within the condensate. Accordingly, the main rationale for the high magnitude of the fluorescent tail and the efficient proton geminate recombination process is that the inner aqueous environment within the condensates facilitates recombination. This scenario will happen when the photoacid is solvated within a nm-scaled microcavity surrounded by proton-reflective boundaries, resulting in the inability of the proton to diffuse away from the *RO* ^–*^, thus, promoting proton recombination. Using a proton diffusion model, Agmon and co-workers have modeled the proton geminate recombination process as a spherical symmetric diffusion problem (SSDP).^45–48^ The SSDP models proton diffusion from the photoacid for distances of *a* < *r* < *r*_max_, where *a* is the contact radius for the photoacid, i.e., the physical dimensions of the probe (*a* was 5.5 Å in our calculations), *r* is the radius of proton diffusion, and *r*_max_ represents a maximum radius for proton diffusion (beyond this value, the proton cannot diffuse anymore). The latter value can be infinity for unrestricted proton diffusion, such as for a solvated photoacid in solution, but, in practice, any value greater than 15 nm yields an identical curve. A value of *r*_max_ below 15 nm suggests the presence of a proton-reflective boundary, which enhances geminate proton recombination. This is valid as long as the proton cannot recombine with the boundary itself (hence, its reflective nature. By fitting the time-resolved fluorescence of photoacids within the SSDP model, the time-resolved decay of photoacids can be used to extract *r*_max_. This model was used to estimate the confined nm-scaled cavities within reversed micelles or peptide-based hydrogels.^31,32^ We used this model, which is available through the SSDP program, to fit the obtained fluorescence decay. For convenience, Figure 3 presents several simulated graphs illustrating how the change in the maximum radius (*r*_max_) of the confined aqueous nanostructure, compared to the decay of HPTS in the polyK-UTP condensates. The smaller the radius, the greater the chance of geminate recombination, i.e., the higher the fluorescence tail. Importantly, since geminate recombination can happen only after the ESPT process, the initial decay is identical no matter what *r*_max_ is. This effect of the confined space on the radiative decay is substantially different from the decay in restricted dimensionality systems, which manifests in the slope of the tail (Figure S7a). Importantly, a change in either the size of the confined space (*r*_max_) or the dimensionality (*d*) is needed to account for the high-magnitude fluorescence tail at these time scales (>3 ns), as a change in just the proton recombination parameter (*k*_*a*_) cannot recapitulate the kinetics of the fluorescent decay, and it manifests primarily at <3 ns time scales) (Figure S7b). We have simulated, using the SSDP program, the decay of HPTS within the condensates to extract several important parameters.

**Figure 3:**
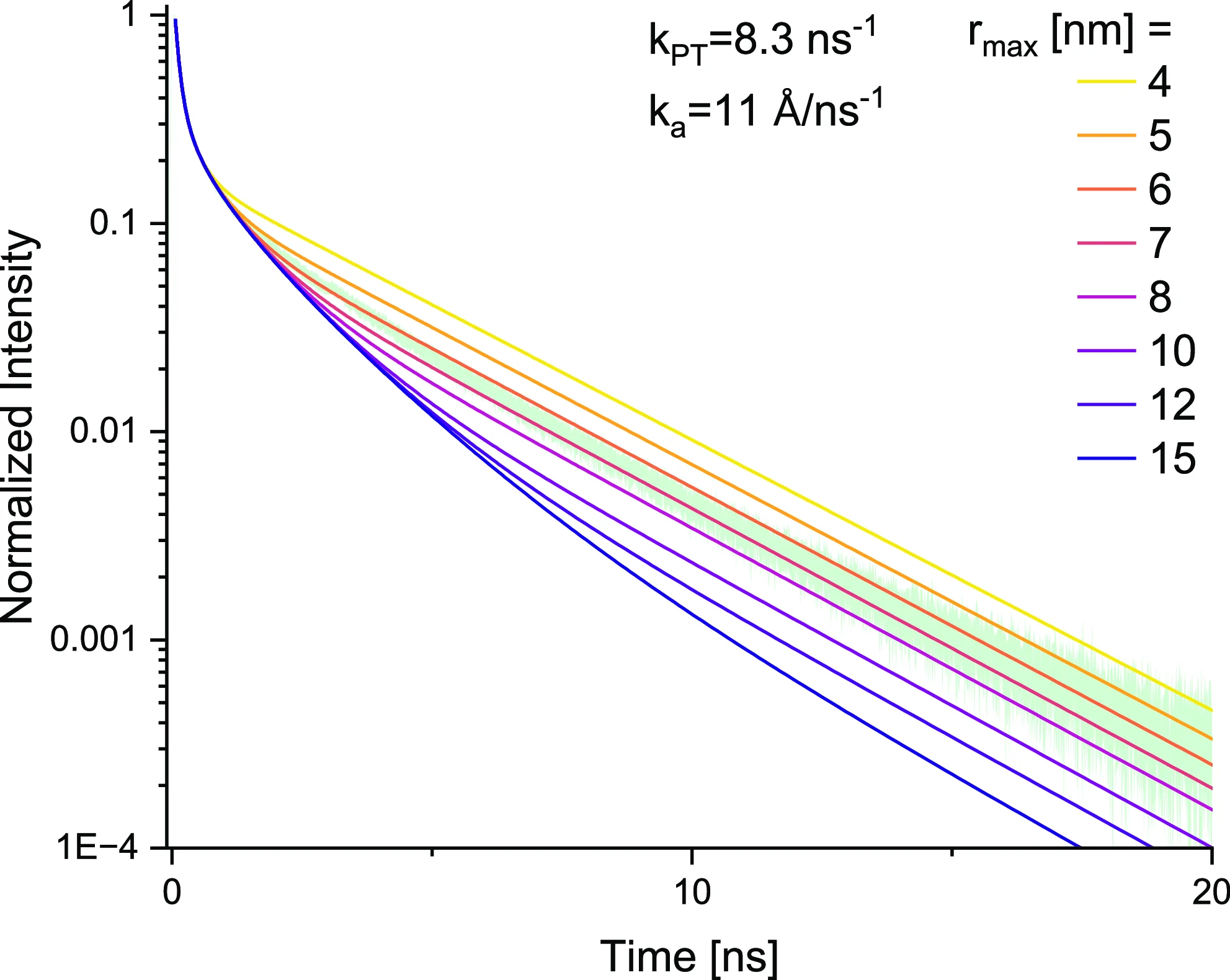
A simulation for the fluorescence decay of HPTS (ROH*) using the SSDP program shown alongside the decay of HPTS in polyK-UTP condensates (the green shaded area, which represents the standard deviation, Figure 2a). All the simulated curves share an ESPT rate of *k*_PT_ = 8.3 ns^–1^ and a proton recombination rate of in *k*_*a*_ values of 11.0 Å/ns^–1^, while the *r*_max_ of the confined water nanocavity within the condensates was changed.

The first is *k*_PT_, which is slightly slower for HPTS within the condensates compared to solvated HPTS in the bulk. The fitting results in *k*_PT_ values of 8.3 ± 0.1 ns^–1^. In comparison, the *k*_PT_ value of solvated HPTS in the buffer condition used in this study is ∼10 ns^–1^ (Figure S8). The slightly lower ESPT efficiency of HPTS within the biomolecular condensates resonates with the described earlier works using other environmental fluorophores, which show that the water phase within the condensates is more viscous than bulk, resulting in decreased water dipolar relaxation. As shown in previous studies with HPTS, such an environment can result in a slower ESPT process.^45^

The second is *k*_*a*_, which is the proton geminate recombination process. As discussed, we claim that the nanocavity formed by water pools within the condensate leads to increased proton recombination owing to the confinement of the biophysical fluorescent probe. In the SSDP model, *k*_*a*_ is not a simple kinetic term as *k*_PT_, but it is a surface reactivity parameter that represents the local linear rate at which a proton crosses the reaction boundary once it reaches the contact distance *a* during the proton recombination process. Indeed, the fitting results in larger *k*_*a*_ values of 11.0 ± 0.7 Å/ns^–1^ for HPTS within the condensates, compared to the value of 4.5 Å/ns^–1^ for solvated HPTS in the buffer conditions (Figure S8).

The next and most important parameter is the maximum radius, *r*_max_, of the inner aqueous volumes (the nanocavity) within the biomolecular condensate. This term can be referred to as the outer sphere of the photoacid, representing a boundary condition, where the dissociated geminate proton cannot penetrate it, nor stick to it, hence, it is a reflective boundary (i.e., promotes proton recombination). The best fittings were recorded with an average *r*_max_ value of 6.5 ± 0.6 nm. This value does not mean perfect water-filled spheres within the biomolecular condensates. Biomolecular condensates are dynamic systems with their inner structure constantly reshaping. Accordingly, this maximum distance value represents an average distance maximum for proton diffusion within the suggested cavities. Nonetheless, this value is one of the main impacts of this work and represents a unique capability of photoacids that is absent for other molecular fluorophores.

The last parameter we need to discuss is the proton diffusion dimensionality (*d*). While we did not expect a change in *d*, the decays could not be fitted without slightly lowering the *d* values of proton diffusion to 2.7. The reduction in d, together with the low volume of the nanocavity, indicates that the microstructure is not perfectly spherical, which is a reasonable outcome. In a confined nonperfectly spherical volume, the proton diffusion will have some directionality. Another possible reason for the reduction in *d* values is the electrostatic attraction of the HPTS probe to the components of the biomolecular condensates, which create the confined microenvironment. While we established that the photoacid is well solvated and can undergo ESPT, its proximity to the “walls” of the confined microenvironment can also reduce dimensionality, as shown, for instance, with the absorption of HPTS to other biological surfaces.^28^

To further confirm the solvation of HPTS within the biomolecular condensates, which is related to their ESPT capability, we performed fluorescence anisotropy measurements of the ESPT process (Figure S9), focusing on the ${{ROH}^{*}/RO}^{-*}$ ratio rather than overall fluorescence intensity (due to fluorescence scattering in some samples). If the photoacid is absorbed into a surface, it will show a large anisotropy for its ${{ROH}^{*}/RO}^{-*}$ ratio, while a well-solvated photoacid will not exhibit substantial fluorescence anisotropy owing to its free rotation in solution. As shown in Figure S9, HPTS within the PolyK-UTP condensates does not exhibit large fluorescence anisotropy (unlike the other condensates in this study, further details below).

The fluid and amorphous nature of the biomolecular condensate, along with the observed reduced proton diffusion dimensionality, imply that the average nanocavity maximum radius serves as an effective confinement length scale. As such, these nanocavities are not necessarily spherical, as was observed in reverse micelles,^31,32^ but can be more of a percolating network, as was observed in peptide-based hydrogels.^31,32^ It is worth highlighting that the fitted curves are averaged curves taken for a large ensemble of condensates present in solution (not a single condensate experiment). Accordingly, the extracted values represent averaged values of a heterogenic population. While comparing the extracted maximum radius to the extracted mesh size radii within biomolecular condensates using other techniques and simulations, our extracted values can be placed at the larger size, but well within the mesh size of common condensates, where even larger radii were observed.^13,49,50^

### The Role of the Biomolecular Condensate Composition on the Inner Water Phase

In the last part of our study, we will explore how the composition of biomolecular condensates influences the biophysical properties of their inner water phase. As discussed above, the nucleobase acts as an anionic bridge that connects the positively charged lysine residues and drives LLPS and biomolecular condensate formation. Taking into consideration the differences in the molecular structure of the adenine purin base and the uracil pyrimidine base, the ATP-containing condensates are denser and have a more hydrophobic interior (i.e., less hydrated), which is stabilized by stronger π–π and hydrogen-bond interactions than UTP-containing condensates.^35^ Replacing polyK with polyR has an even more dramatic effect on the internal structure of the condensate. Owing to the presence of the guanidinium group in polyR instead of the α-amino group in polyK, the polyR-based condensates are much denser, hence viscous, which is consistent with their stronger multivalent interactions with the nucleotide partner.^33^

Here, we used our fluorescent probes to explore how the composition of the biomolecular condensate influences the water phase within it. Unlike the polyK-UTP system, we used only the HPTS photoacid for the condensates in this section: polyK-ATP and polyR-UTP. Because of the low fluorescence quantum efficiency of 6AQ compared to HPTS and to the charge of the molecule, we could not confirm the successful encapsulation of the 6AQ in the condensates here, which are more aggregative than the polyK-UTP system.

We first verified using fluorescence microscopy that the photoacid was integrated into the condensates (Figure 4a,b for polyK-ATP and polyR-UTP), where a similar probe partition as for the polyK-UTP condensates was estimated. Replacing UTP with ATP in the polyK-based condensate resulted in a relatively mild change in the steady-state fluorescence (Figure 4c). Nonetheless, it is clearly evident that the ROH* band in the steady-state fluorescence is higher within the polyK-ATP condensates than in the polyK-UTP ones. We also verified that the observed change is not due to a change in the p*K*_a_ of HPTS owing to the different environment, as evidenced by the fluorescence excitation spectrum, which shows a rather similar excitation spectrum for HPTS across all condensates used here and for free solvated HPTS (Figure S10). To understand whether that change is because of a slower ESPT process (lower *k*_PT_) or a more efficient proton recombination process (larger *k*_a_), we turned to time-resolved fluorescence (Figure 4d). As seen in the transients, the initial decay over the first 0.5 ns is slower in the polyK-ATP condensates, and the magnitude of the long-lived fluorescence tail is higher in these condensates. These observations indicate that both the ESPT is slower and the geminate recombination process is more efficient in the polyK-ATP condensates than in the polyK-UTP ones. For a more quantitative estimation, we turned again to the SSDP program. Fitting the transient of the polyK-ATP condensates resulted in values of: *k*_PT_ = 7.3 ± 0.3 ns^–1^, *k*_a_ = 11.6 ± 0.9 Å/ns^–1^, and *r*_max_ = 5.8 ± 0.8 nm. As for the polyK-UTP condensates, we had to reduce the dimensionality of proton diffusion to 2.7. The slower ESPT process within the polyK-ATP condensates can be attributed to the higher viscosity within them. The slightly faster proton recombination rate and the smaller nanocavities within the polyK-ATP condensates are indicative of a denser configuration. In line with these observations, the fluorescence anisotropy within the polyK-ATP condensates is also slightly larger than in the polyK-UTP condensates (Figure S9).

**Figure 4:**
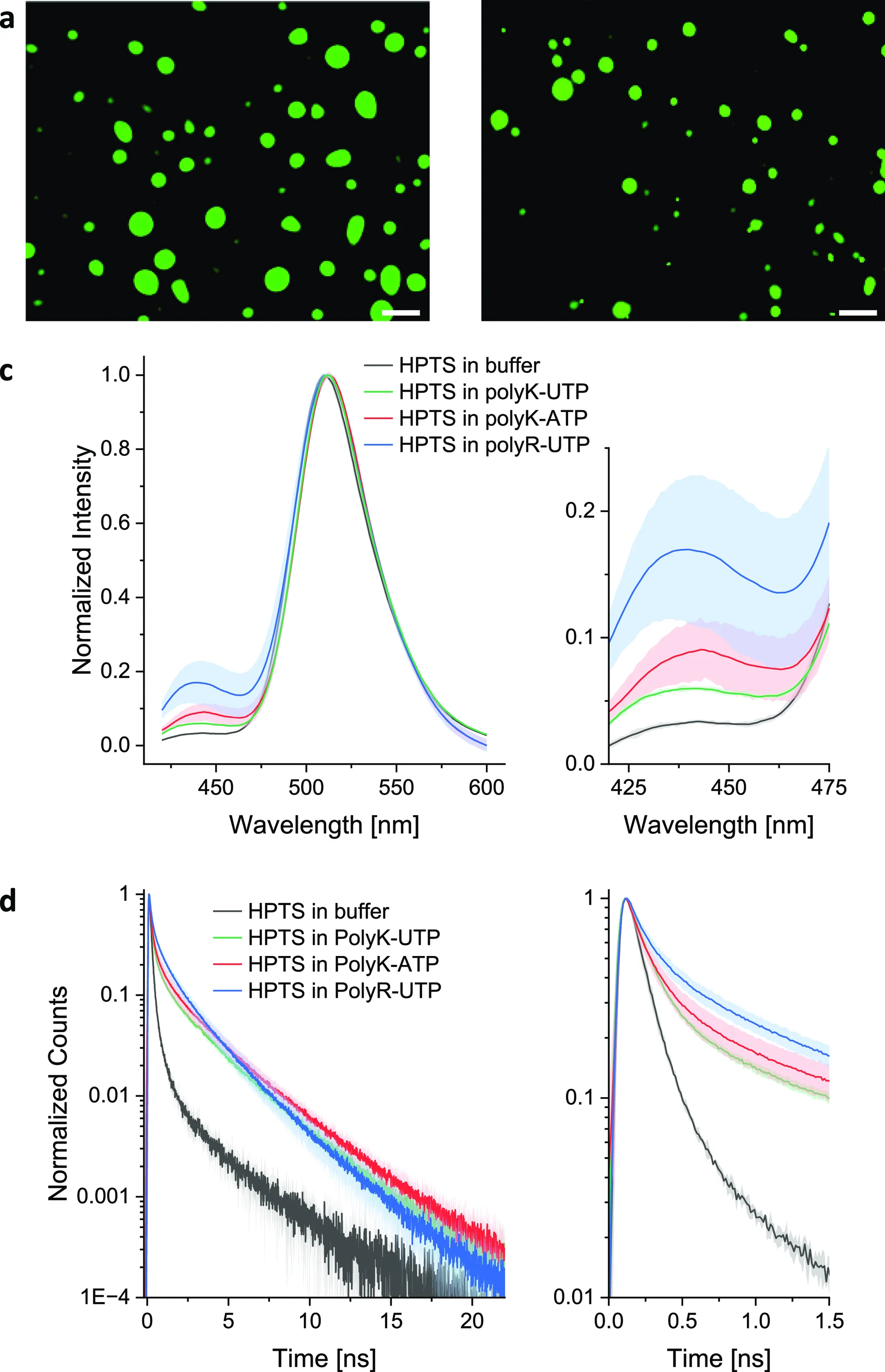
Confocal microscopy images of HPTS within (a) polyK-ATP and (b) polyR-UTP condensates. The scale bar represents 10 μm. (c) Steady-state fluorescence and (d) time-resolved fluorescence (at the position of ROH* at 440 nm) of HPTS within all the biomolecular condensates studied in this study compared to solvated HPTS in the same buffer. The shaded area represents the standard deviation of *N* = 3 for HPTS in buffer and *N* = 6 for HPTS in the condensates. The right panels are the zoom-in section of the steady-state ROH* band (for panel (c)) and of the first 1.5 ns of the decay (for panel (d)).

The most dramatic effect was observed while replacing polyK with polyR (thus making a polyR-UTP condensate). As seen in the steady-state spectra (Figure 4c), the ROH* band magnitude of the polyR-UTP condensates is higher than the polyK-UTP ones. While the excitation spectrum of HPTS within these condensates is similar to the others (Figure S10), there is a noticeable shift in the peak position, which already indicates a difference in the solvation properties of HPTS within polyR-UTP condensates. As before, to better understand whether the change in the steady-state spectrum is due to a change in the initial ESPT process or the subsequent proton recombination process, we turned to time-resolved fluorescence (Figure 4d). The time-resolved fluorescence clearly shows that the initial decay of the polyR-UTP condensates is considerably slower than in the polyK-UTP condensates (right panel of the figure). Importantly, the slope of the decay is also fundamentally different from that in the polyK-UTP condensates, resulting in a lower-magnitude fluorescence tail. While for the polyK-based systems, the fluorescent transient was indicative of a solvated photoacid in a confined microcavity (high *k*_PT_ and high *k*_a_), the transient in the polyR-based system is indicative of a situation where the photoacid is not fully dissolved, resulting in a low ESPT rate (*k*_PT_) while still maintaining a relatively high proton recombination (*k*_a_). The low ESPT rate (corresponding to the initial fluorescence quenching) is directly related to the solvation of the photoacid. Accordingly, this finding already suggests that the greater density and water content of the polyR-UTP condensates limit ESPT. The high recombination is related to the poor diffusion of the geminate proton following dissociation. Under these conditions, the photoacid cannot be used as a molecular ruler. Indeed, while fitting the fluorescence decay of HPTS within the polyR-UTP condensates, we extracted the following values *k*_PT_ = 5.1 ± 0.2 ns^–1^, *k*_a_ = 10.3 ± 0.8 Å/ns^–1^, with no restriction on *r*_max_. The latter parameter does not mean large microcavities in these condensates, but the lack of reflecting boundary conditions for a diffusive geminate proton. Overall, our results with the polyR-UTP condensates clearly demonstrate that the inner water phase within them is very different from that in polyK-based condensates. The water phase within the polyR condensates does not allow a full solvation of the HPTS molecule. Most probably owing to electrostatic interactions between the fluorophore and the polyR peptide. Indeed, the fluorescence anisotropy of HPTS within these condensates is the highest among all other condensates (Figure S9).

## Conclusions

In summary, we demonstrated the use of mainly photoacids, but also photobases, as new fluorescent probes to explore the inner water environment of biomolecular condensates, using model condensates of IDPs: polyK or polyR peptides with UTP or ATP. In line with other environmental fluorescent probes, photoacids and photobases are also sensitive to their near environment. However, unlike other fluorophores, they have a different mechanism: the ESPT process and the subsequent proton recombination for photoacids or solvent reorganization for photobases. We showed that the ESPT of both the HPTS photoacid and the 6AQ photobase is only mildly slowed down within the polyK-UTP biomolecular condensates compared to the same process in bulk water. This finding indicates that the probes are well solvated within the polyK-UTP condensates, but the water phase within the condensates remains more viscous, with slower water dynamics than in bulk water. However, we found that the subsequent processes are very different within the condensates compared to water. For the 6AQ photobase, we found a substantially slower solvent reorganization within the condensates, which is consistent with the current understanding of the water phase within such condensates. For the HPTS photoacid, we showed that proton recombination is notably enhanced within the condensates. We attributed the increase in proton recombination to nm-scaled confinement of the dissociated probe within the condensate’s microstructure, resulting in reflecting boundaries of the proton. This finding highlights the unique advantage of using photoacids in biophysical exploration of confined spaces, where, while using established diffusion models, the proton recombination process serves as a molecular ruler to estimate the distance of proton diffusion and, hence, the dimensions of the confined water phase. We further showed how the composition of the biomolecular condensates affects the ESPT-related properties of the photoacid, which are dictated by the water phase and the solvation of the photoacid. We found that replacing UTP with ATP results in a minor yet noticeable slowing of the ESPT process, which indicates a more viscous environment. Accordingly, the dimensions of the confined water phase are also slightly lower in the ATP system than in the UTP system. Replacing polyK with polyR resulted in a much more pronounced effect: a significant reduction in the ESPT rate constant. This finding indicates that the photoacid is less solvated in polyR-based condensates, likely due to stronger electrostatic interactions between the photoacid and the polyR system. It is important to note that the structure of the biomolecular condensate is closely related to which properties can be extracted using photoacids and photobases that can be partitioned into the condensed phase. Here, we use polyK/polyR systems that are not expected to capture the dissociated proton, and accordingly, photoacids can be used as molecular rulers. However, for different biomolecular condensates with varying compositions, this scenario can differ. Nonetheless, the use of photoacids and photobases can be important both for understanding how different drugs are solvated within biomolecular condensates and for biophysical exploration of various biomolecular condensate systems.

## Materials and Methods

### Materials

Poly­(l-lysine hydrochloride) (*M*_W_ = 1600 Da, *N* = 10, DP_n_ determined by NMR = 8–12) and poly­(l-arginine hydrochloride) (*M*_W_ = 1900 Da, *N* = 10, DP_n_ determined by NMR = 8–12) were purchased from Alamanda Polymers (USA) and used as received. Uridine 5′-triphosphate (UTP, trisodium salt) was obtained from Chemscene, and adenosine 5′-triphosphate (ATP, disodium salt hydrate) was obtained from Sigma-Aldrich. 8-Hydroxypyrene-1,3,6-trisulfonic acid (HPTS) was purchased from Thermo Scientific, and 6-aminoquinoline (6-AQ) was obtained from Alfa Aesar. HEPES was purchased from Bio-Lab. Nuclease-free water was used for all preparations.

### Stock Solutions Preparation

A 50 mM HEPES buffer solution was prepared and adjusted to pH 7.1 with sodium hydroxide (NaOH). Stock solutions of ATP and UTP were prepared at a concentration of 100 mM. Poly­(l-lysine) (polyK) and poly­(l-arginine) (polyR) were prepared at 50 mg/mL in nuclease-free water and stored at 4 °C. Prior to use, polymer solutions were sonicated according to the manufacturer’s instructions. Additionally, 1 mM stock solutions of HPTS and 6-AQ were prepared.

### Condensate Preparation

Condensates were formed via charge neutralization between polycation and polyanion components. Samples contained 6 mM polycation (either poly-l-lysine or poly-l-arginine, calculated per monomer assuming one positive charge per repeat unit) and 1.45 mM UTP or ATP in 50 mM HEPES buffer (pH 7.1). At this pH, each nucleotide carries approximately four negative charges, corresponding to the chosen ratio for near charge neutrality.^33^ Hybrid condensates were obtained by allowing the mixtures to equilibrate for 30 min, followed by the addition of 5 μM HPTS. Due to its four negative charges, HPTS compensated for the slightly reduced nucleotide content and preferentially partitioned within the condensate phase.^51^ A similar procedure was used to prepare hybrid condensates containing 10 μM 6-aminoquinoline (6-AQ), where the polycation concentration was slightly reduced to 5.88 mM to maintain charge balance with 1.5 mM UTP or ATP.

### Microscopy Imaging

Fluorescence imaging of HPTS-containing condensates was performed using a Leica DMI8 inverted fluorescence microscope equipped with a 100× oil-immersion objective (NA 1.4) and a 2.8 MP color CCD camera. Image acquisition and analysis were carried out using Leica LAS X software. Fluorescence imaging of 6-AQ-containing condensates was performed using a Zeiss LSM 710 inverted confocal microscope equipped with a 63× oil-immersion objective. Images were acquired using a PMT detector and processed with Zeiss ZEN software. Samples were mounted on μ-Slide coverslip chambers (Ibidi) and imaged in fluorescence mode.

### Time-Resolved and Steady-State Fluorescence Measurements

Steady-state fluorescence measurements were performed using either an FS5 (Edinburgh Instruments) or a Fluorolog (Horiba) spectrofluorometers. The excitation wavelength was set to 405 nm for HPTS and 350 nm for 6-AQ. Time-correlated single-photon counting (TCSPC) measurements were carried out using a CHIMERA spectrometer (Light Conversion) with an excitation wavelength of 405 nm for HPTS and 350 nm for 6-AQ. The excitation source was a 1030 nm, 10 W Yb-based PHAROS laser (Light Conversion), operating at a repetition rate of 1 MHz, delivering pulses of 190 fs duration and a maximum pulse energy of 10 μJ. The laser output was directed through an optical parametric amplifier (ORPHEUS, Light Conversion), and the scattered excitation beam was subsequently coupled into the CHIMERA spectrometer. Time-resolved fluorescence signals were detected using a hybrid photomultiplier detector (Becker & Hickl, HPM-100-07) operated in single-photon counting mode. The instrument response function (IRF) was approximately 50 ps (full width at half-maximum, fwhm).

## Supplementary Material



## Notes

Table of molecular schemes of the molecules used in this study; additional bright-field microscopy images of the condensates; the Förster cycle; additional spectroscopy graphs: steady-state and time-resolved fluorescence of the controls; anisotropy measurement and fluorescence excitation spectrum; and additional simulated time-resolved data (PDF)
