# Supplementary material for: The Hidden Geometry of Water: Mapping Nanoscale Confinement and Water Properties Inside Peptide–Nucleotide Biomolecular Condensates
Source: JACS Au. 2026 Jul 17;6(8):4740–51. doi: 10.1021/jacsau.6c00873 (PMC13508112; doi:10.1021/jacsau.6c00873)
Supplement: Supplementary file 1 [file au6c00873_si_001.pdf]

Supporting Information

The Hidden Geometry of Water: Mapping Nanoscale Confinement and Water Properties Inside Peptide-Nucleotide Biomolecular Condensates

Ayat Bdarneh[1] and Nadav Amdursky[1,2,*]

[1]Schulich Faculty of Chemistry, Technion – Israel Institute of Technology, Haifa, 3200003, Israel

[2]Chemistry, School of Mathematical and Physical Sciences, University of Sheffield, Sheffield S3 7HF, United Kingdom

*Corresponding author: n.amdursky@sheffield.ac.uk

Supplementary Table

Table S1. Molecular structures of polypeptides, nucleotides, photoacid, and photobase used in this study.

Polypeptides		Nucleotides	
Poly-L-lysine	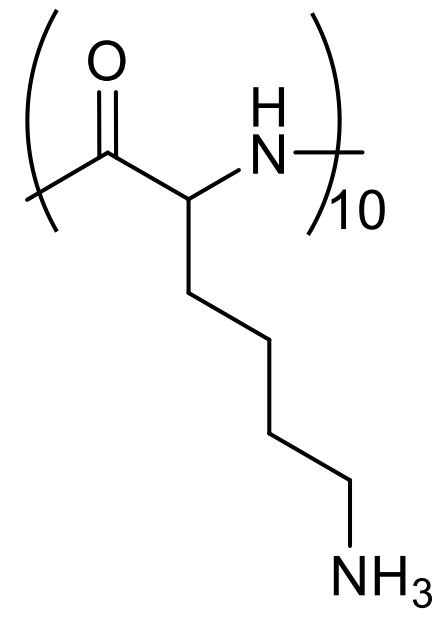	UTP	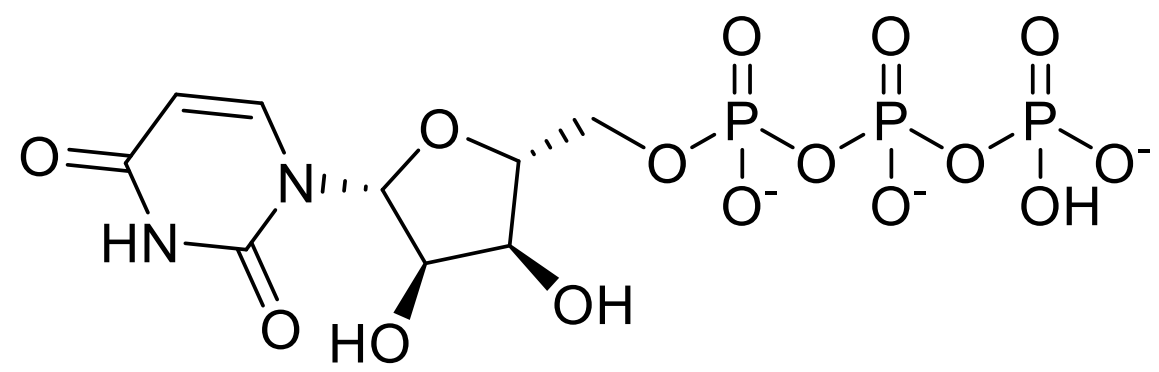
Poly-L-Arginine	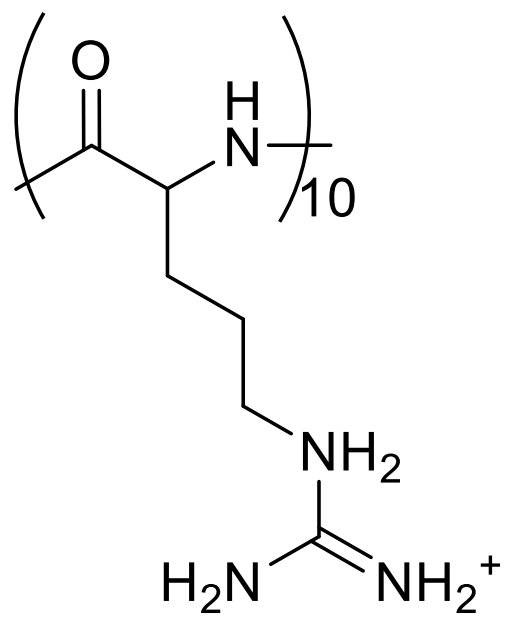	ATP	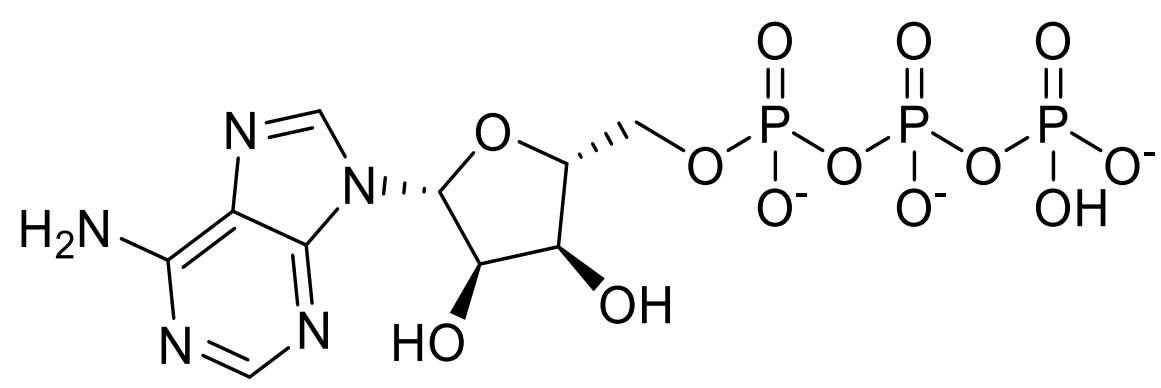
**Photobase**		**Photoacid**	
6-AQ	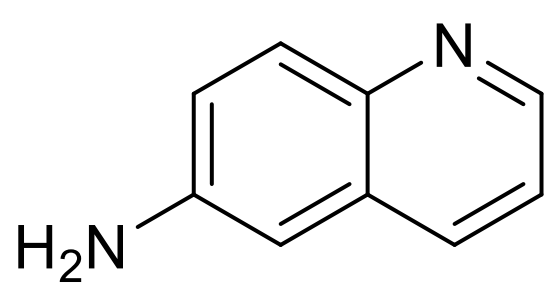	HPTS	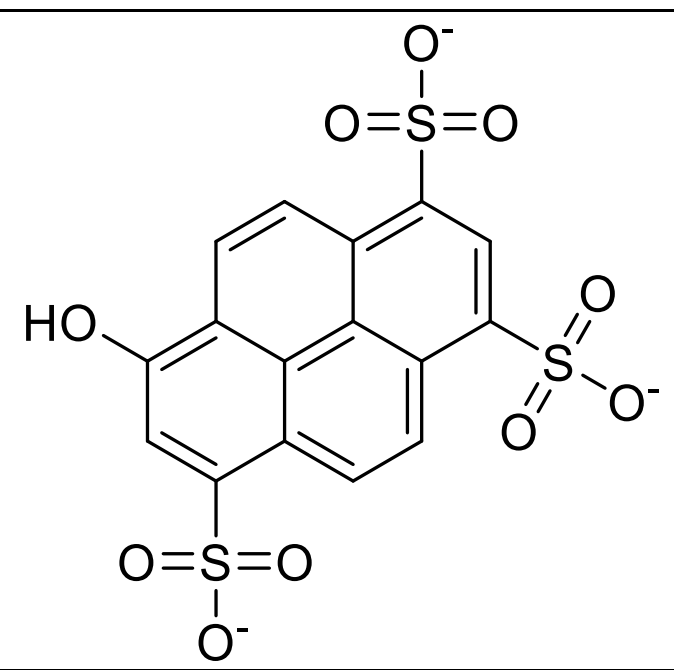

Supplementary Figures

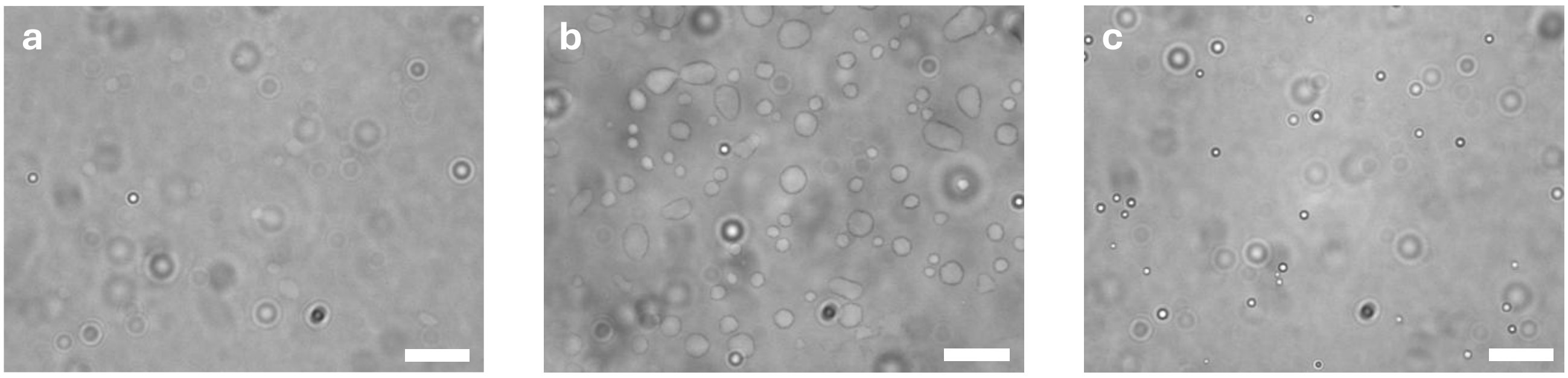

Figure S1. Microscopy images of the biomolecular condensates used in this study: (a) polyK-UTP, (b) polyK-ATP, and (c) polyR-UTP. The scale bar represents 15 µm.

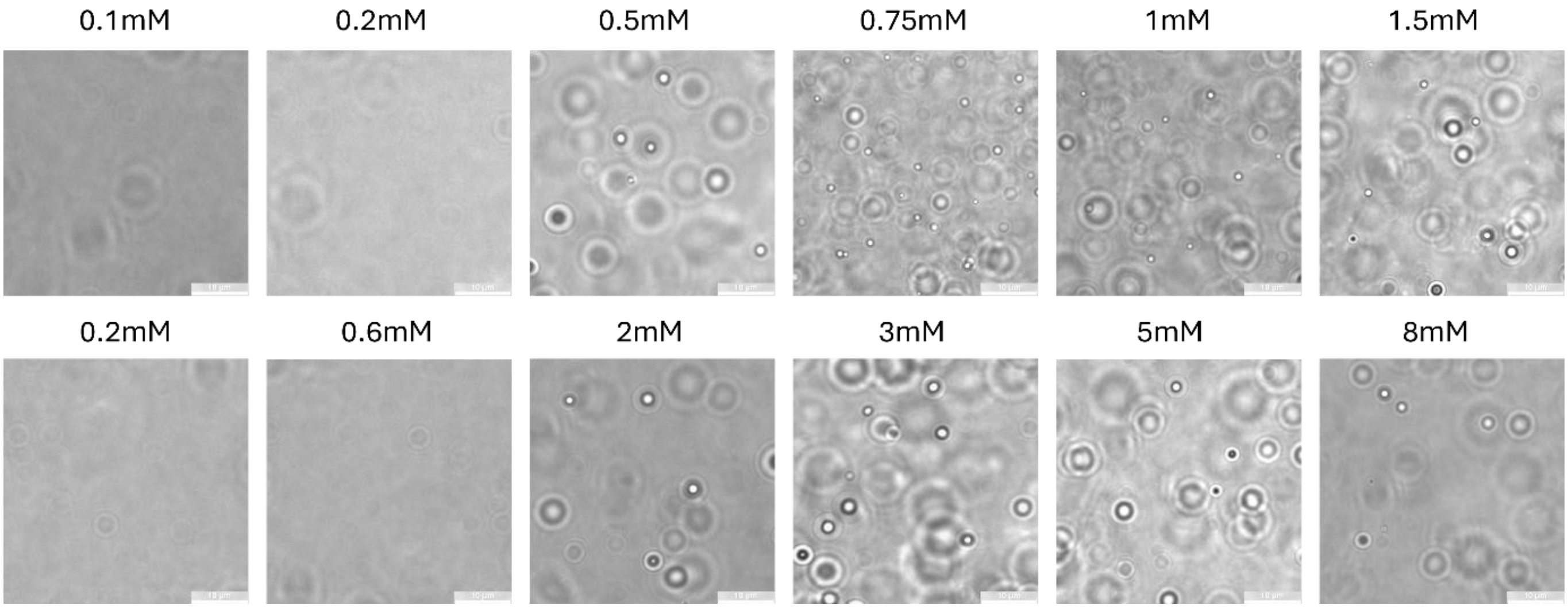

Figure S2. Microscopy images of polyK–ATP condensates formed under varying component concentrations. Top panel: ATP concentration was varied while the polyK concentration was held constant at 6 mM. Bottom panel: polyK concentration was varied while the ATP concentration was held constant at 1.5 mM. Scale bar, 10 µm.

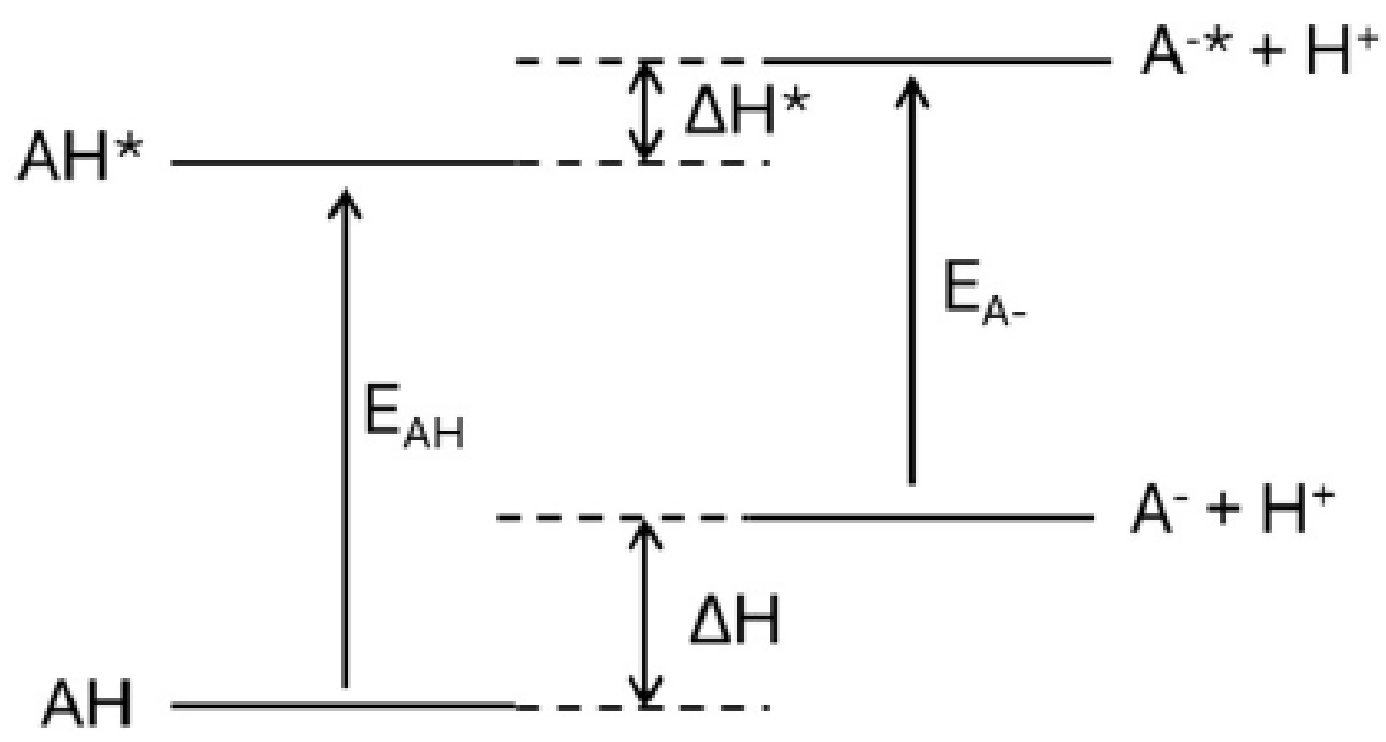

Figure S3. Diagram of the Förster cycle highlighting the difference in energy barrier (corresponds to the acid-base equilibrium) of photoacids between the ground and excited states.

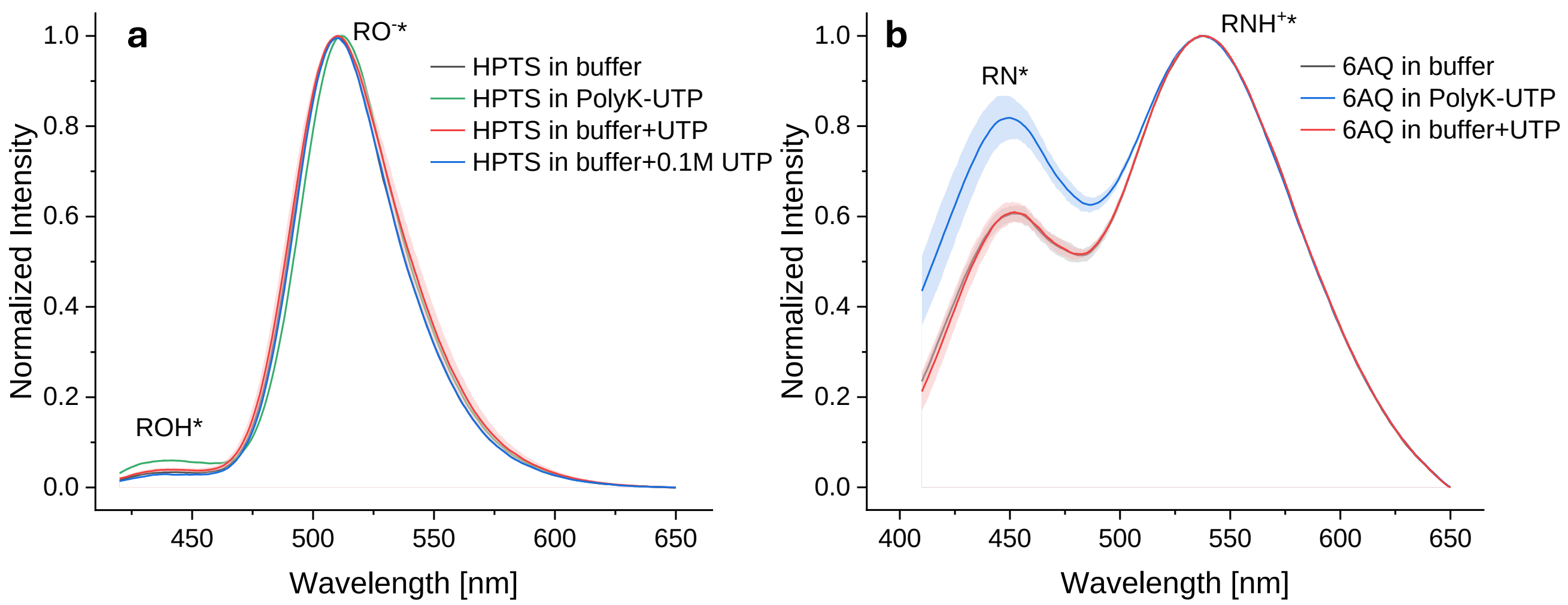

Figure S4. Steady-state fluorescence of (a) HPTS and (b) 6AQ within polyK-UTP condensates compared to solvated HPTS and 6AQ in the same buffer and upon the addition of UTP to the buffer. Panel (a) also shows the spectrum upon the addition of 0.1 M UTP to the buffer (nearly 2 orders of magnitude higher than the concentration of UTP used to make the condensate), which simulates the condensed UTP phase within the condensates.

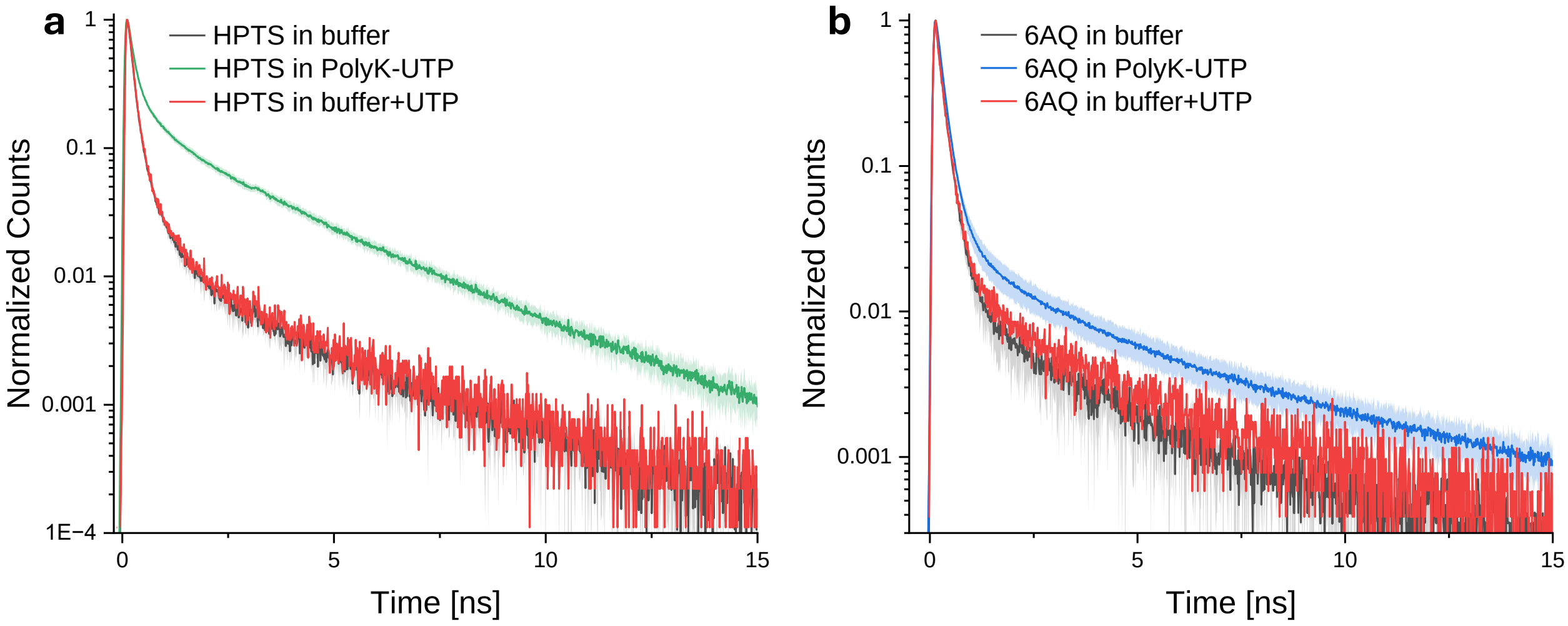

Figure S5. Time-resolved fluorescence of (a) HPTS and (b) 6AQ within polyK-UTP condensates compared to solvated HPTS and 6AQ in the same buffer and upon the addition of UTP to the buffer.

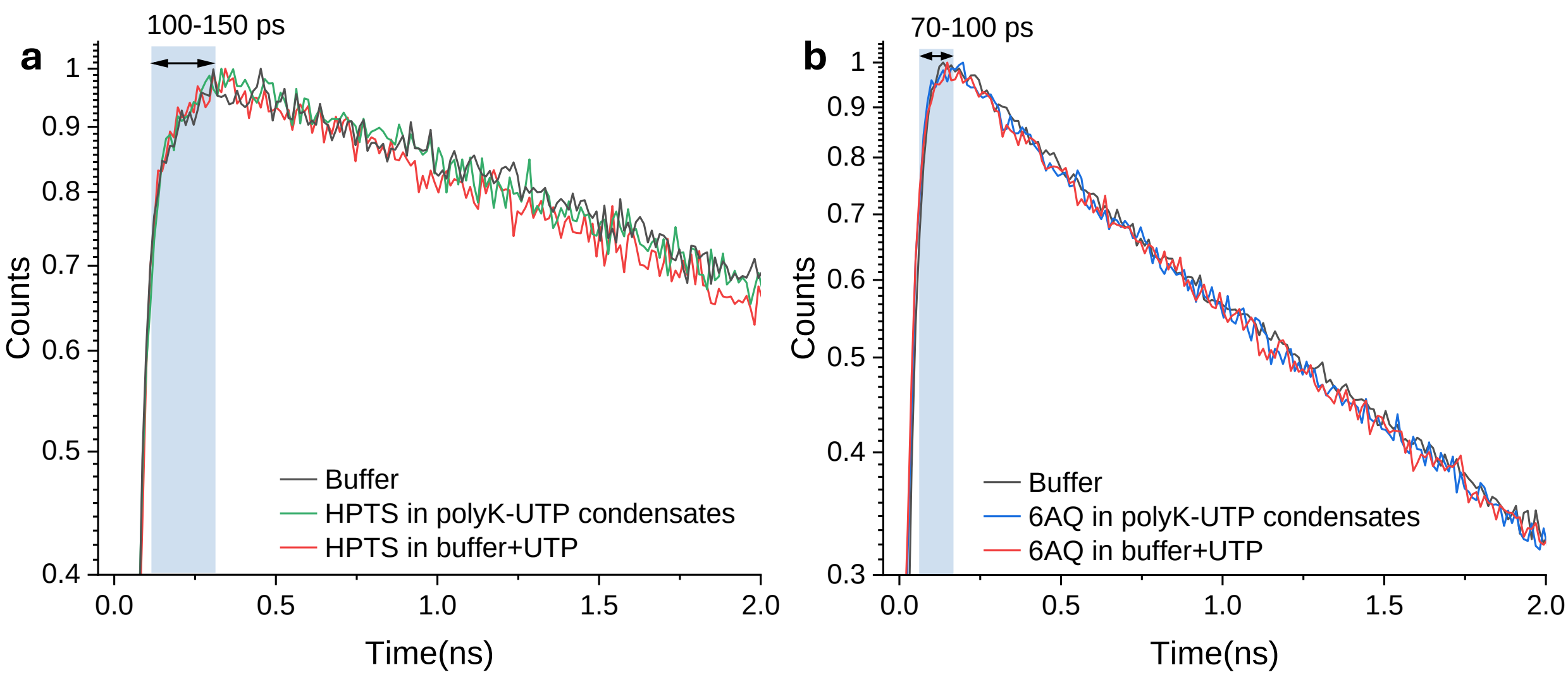

Figure S6. Time-resolved fluorescence of (a) HPTS and (b) 6AQ at the wavelength positions of RO^{-*} and RNH^{+*}, respectively, within polyK-UTP condensates compared to solvated HPTS and 6AQ in the same buffer and upon the addition of UTP to the buffer.

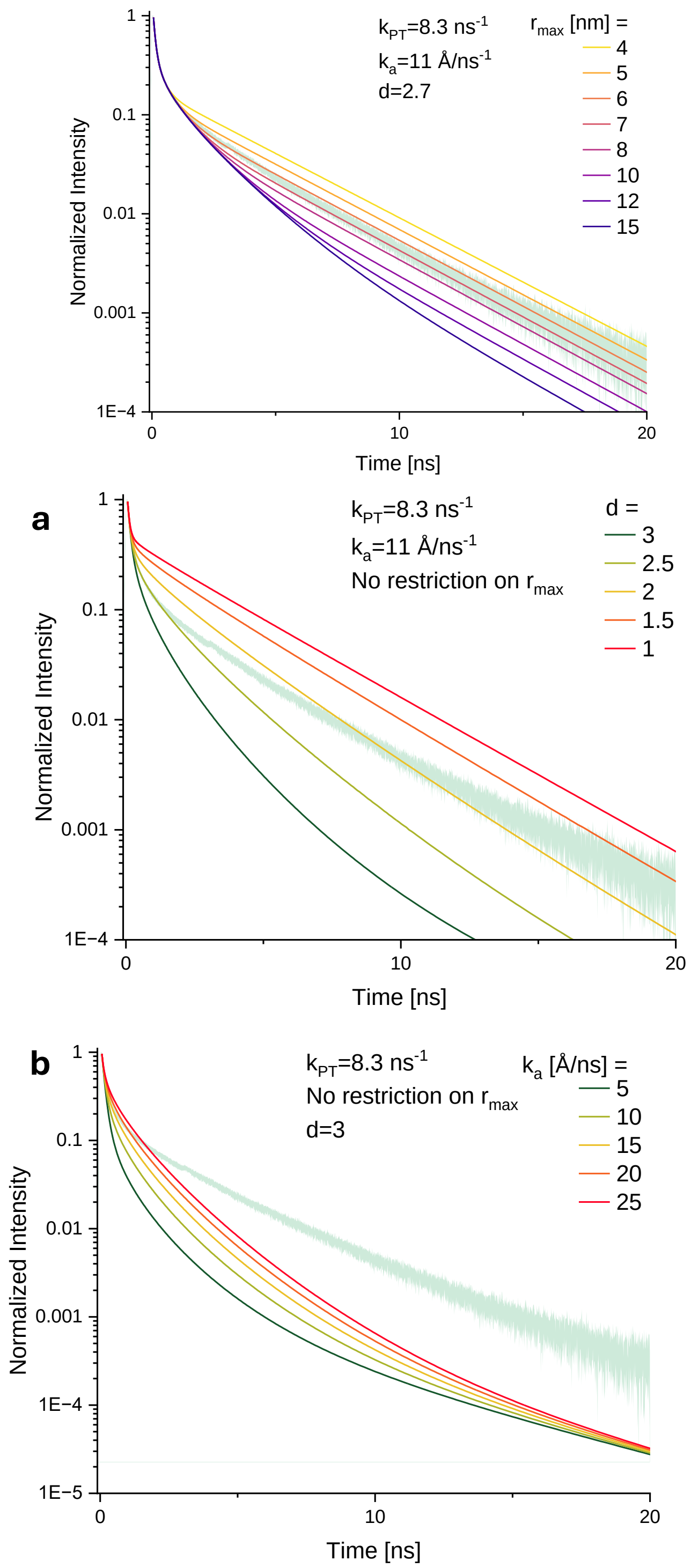

Figure S7. The top panel is Figure 3 in the main text, which shows the simulated decay as a function of the radius (r_{max}) of the confined water nanocavity as a reference. (a) The simulated decay with the same k_{PT} and k_a kinetic parameters, but with no restriction on r_{max} and changing the dimensionality (d) of the proton diffusion from the excited probe. (b) The simulated decay with the same k_{PT} but with varying k_a with no restriction on r_{max} and d (d=3).

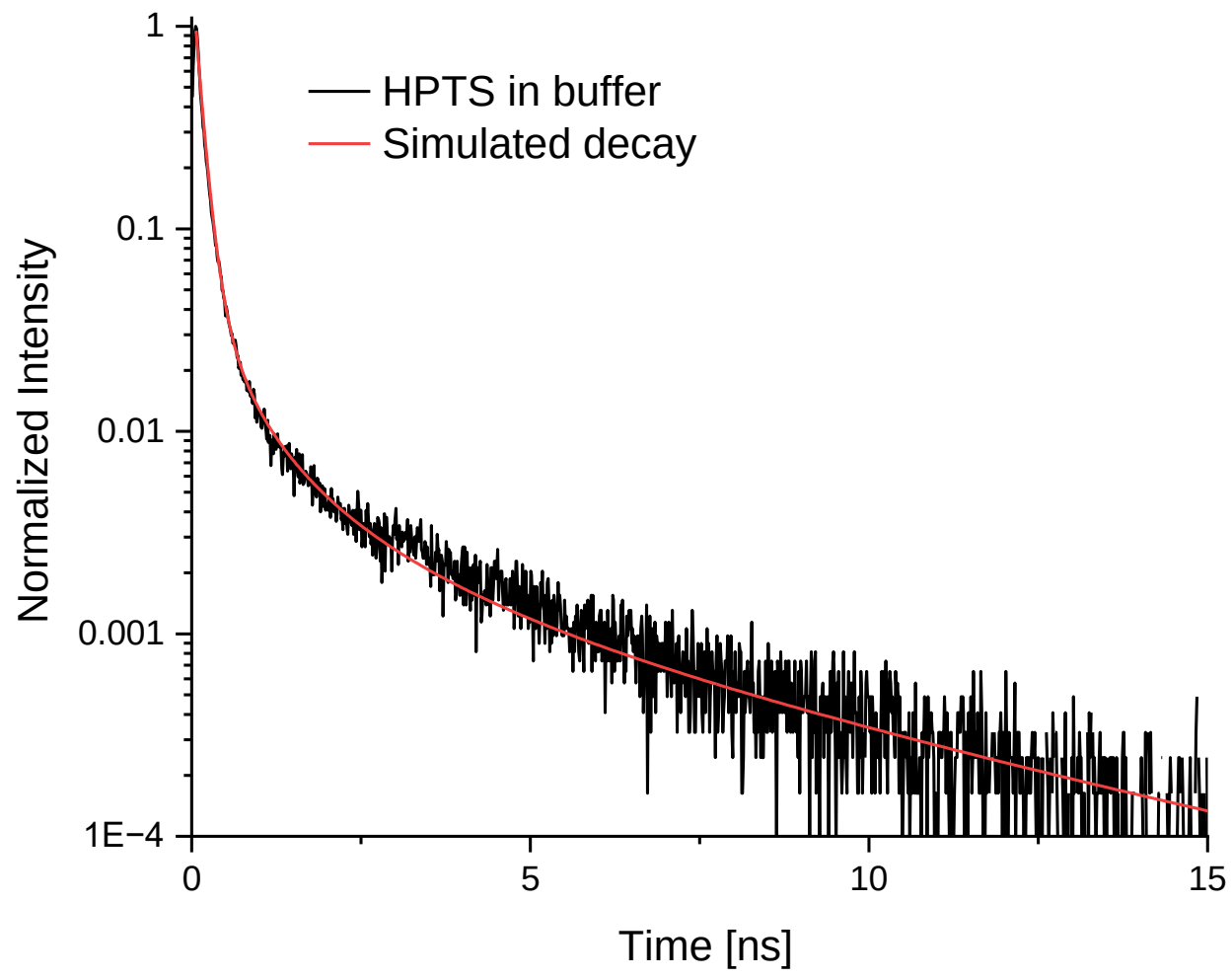

Figure S8. Simulated decay of HPTS in buffer, resulting in the extracted values of: k_{PT}=10 ns^{-1} and k_a=4.5 Å/ns^{-1}.

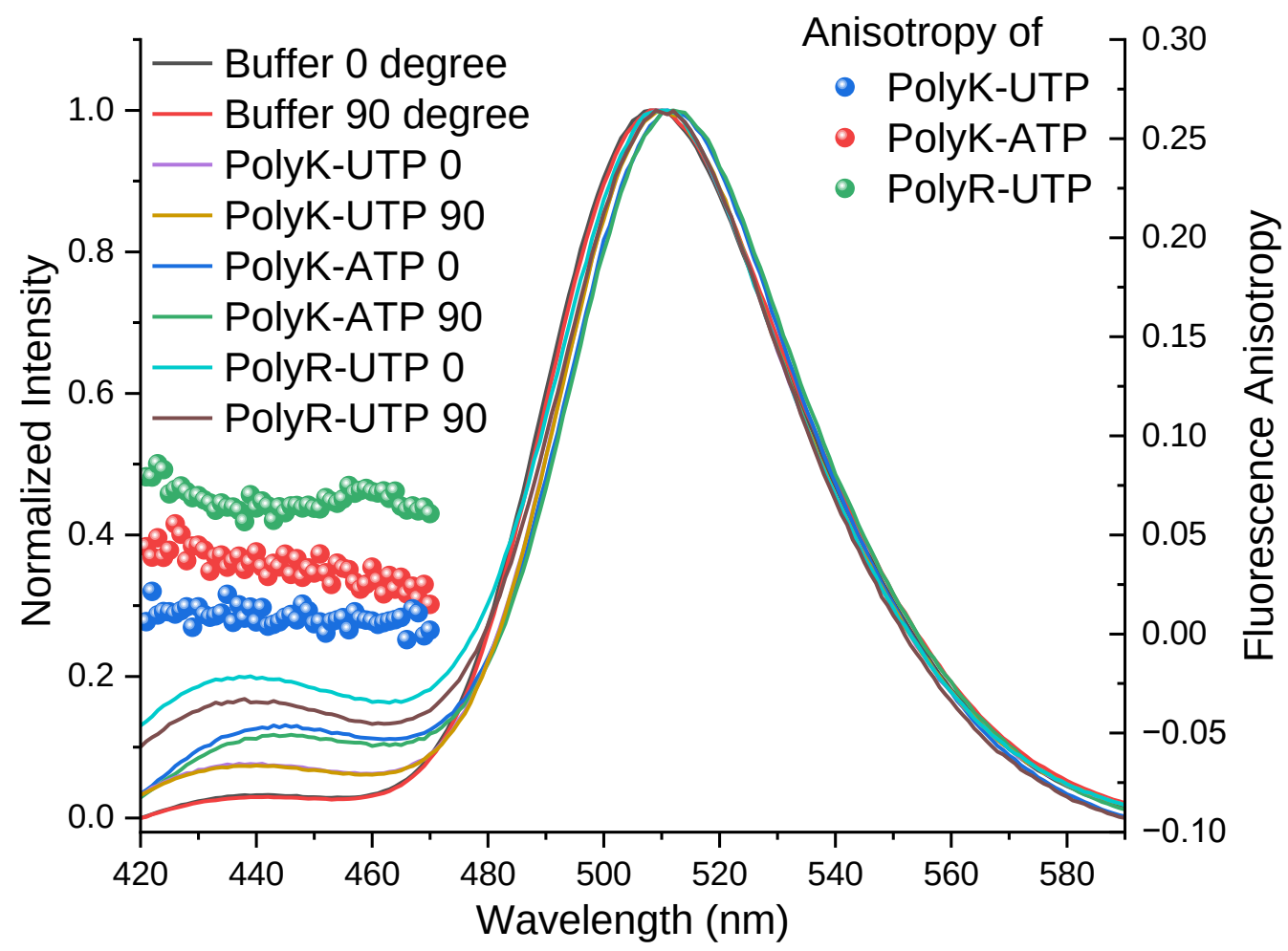

Figure S9. Steady-state fluorescence anisotropy measurements of HPTS in the different investigated systems. The figure shows the curve taken at parallel (0 degrees) and perpendicular (90 degrees) orientations, together with the calculated anisotropy values $((I_{\parallel} - I_{\perp}) / (I_{\parallel} + 2I_{\perp}))$.

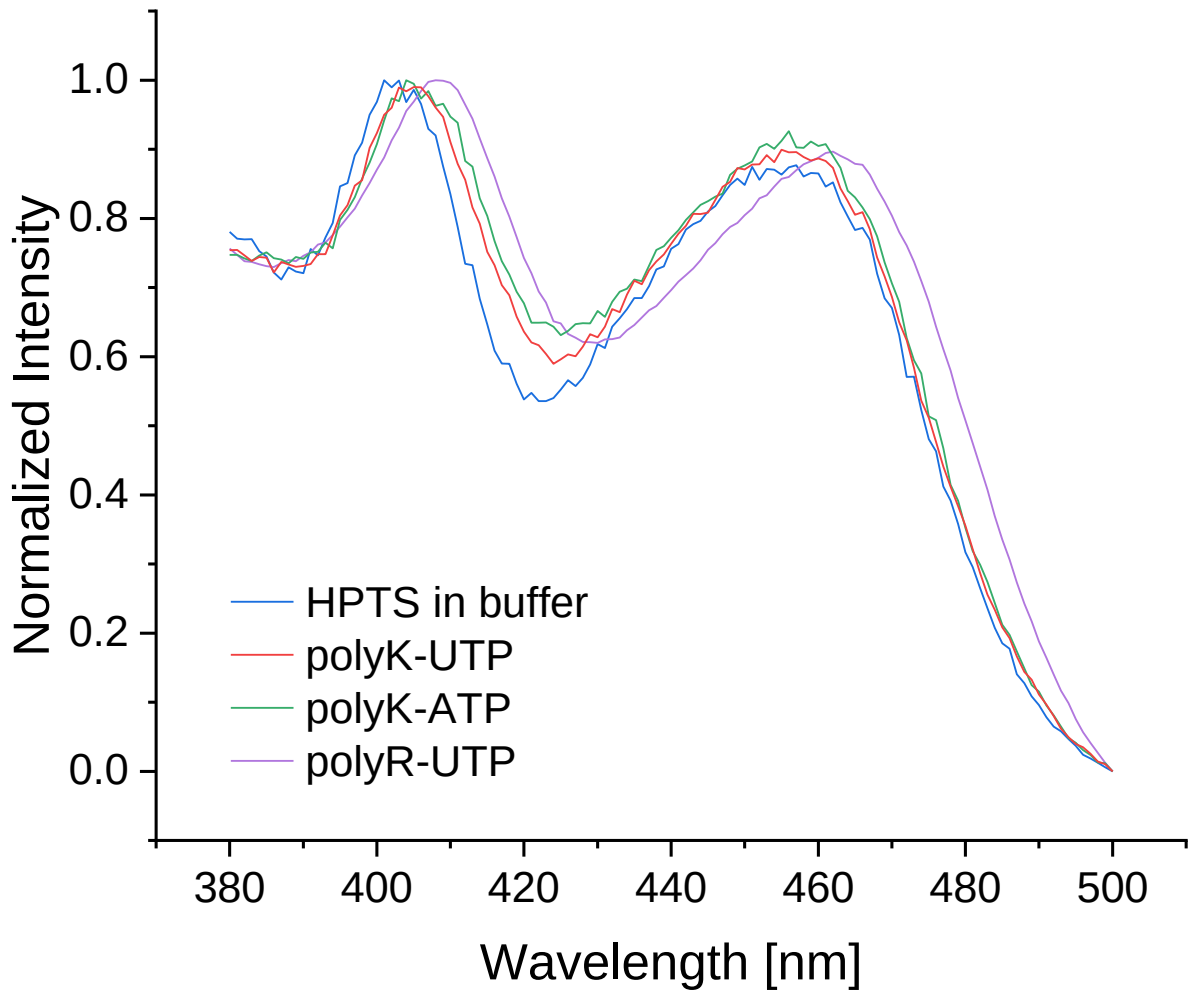

Figure S10. A fluorescence excitation spectrum of HPTS in the different investigated systems.
